# Decreased Expression of Vitamin D Receptors in Neointimal Lesions following Coronary Artery Angioplasty in Atherosclerotic Swine

**DOI:** 10.1371/journal.pone.0042789

**Published:** 2012-08-06

**Authors:** Gaurav K. Gupta, Tanupriya Agrawal, Michael G. Del Core, William J. Hunter, Devendra K. Agrawal

**Affiliations:** 1 Department of Biomedical Sciences and Center for Clinical and Translational Science, Creighton University School of Medicine, Omaha, Nebraska, United States of America; 2 Department of Internal Medicine, Creighton University School of Medicine, Omaha, Nebraska, United States of America; 3 Department of Medical Microbiology and Immunology, Creighton University School of Medicine, Omaha, Nebraska, United States of America; 4 Department of Pathology, Creighton University School of Medicine, Omaha, Nebraska, United States of America; University of Freiburg, Germany

## Abstract

**Background:**

Inflammatory cytokines, such as TNF-α, play a key role in the pathogenesis of occlusive vascular diseases. Activation of vitamin D receptors (VDR) elicits both growth-inhibitory and anti-inflammatory effects. Here, we investigated the expression of TNF-α and VDR in post-angioplasty coronary artery neointimal lesions of hypercholesterolemic swine and examined the effect of vitamin D deficiency on the development of coronary restenosis. We also examined the effect of calcitriol on cell proliferation and effect of TNF-α on VDR activity and expression in porcine coronary artery smooth muscle cells (PCASMCs) *in-vitro*.

**Methodology/Principal Findings:**

Expression of VDR and TNF-α and the effect of vitamin D deficiency in post-angioplasty coronary arteries were analyzed by immunohistochemistry and histomorphometry. Cell proliferation was examined by thymidine and BrdU incorporation assays in cultured PCASMCs. Effect of TNF-α-stimulation on the activity and expression of VDR was analyzed by luciferase assay, immunoblotting and immunocytochemistry. *In-vivo*, morphometric analysis of the tissues revealed typical lesions with significant neointimal proliferation. Histological evaluation showed expression of smooth muscle α-actin and significantly increased expression of TNF-α in neointimal lesions. Interestingly, there was significantly decreased expression of VDR in PCASMCs of neointimal region compared to normal media. Indeed, post-balloon angioplasty restenosis was significantly higher in vitamin D-deficient hypercholesterolemic swine compared to vitamin D-sufficient group. *In-vitro*, calcitriol inhibited both serum- and PDGF-BB-induced proliferation in PCASMCs and TNF-α-stimulation significantly decreased the expression and activity of VDR in PCASMCs.

**Conclusions/Significance:**

These data suggest that significant downregulation of VDR in proliferating smooth muscle cells in neointimal lesions could be due to atherogenic cytokines, including TNF-α. Vitamin D deficiency potentiates the development of coronary restenosis. Calcitriol has anti-proliferative properties in PCASMCs and these actions are mediated through VDR. This could be a potential mechanism for uncontrolled growth of neointimal cells in injured arteries leading to restenosis.

## Introduction

Percutaneous coronary intervention is a common strategy to treat coronary artery disease but, neointimal hyperplasia with resultant restenosis following interventional procedure remains the major limitation [1], [2]. Although the magnitude of intimal hyperplasia and late luminal loss have been significantly reduced by implantation of drug eluting stents (DES), late stent thrombosis requiring longer periods of anti-platelet therapy is a potentially fatal complication [3]. Neointimal hyperplasia, a cell proliferation and differentiation process, is the predominant mechanism in the development of in-stent restenosis and anti-proliferative drugs in DES suppress tissue re-growth [2]. Inflammatory mediators in the atheromatous tissue potentiate the underlying cellular response in the development of restenosis [4]. Indeed, the inflammatory reaction is more prominent after stent implantation compared to balloon angioplasty [2]. Tumor necrosis factor (TNF)-α, a pluripotent pro-inflammatory cytokine, plays a pivotal role in restenosis after coronary intervention [5]. There is marked increase in tissue expression of TNF-α due to arterial injury following balloon angioplasty [6].

Vitamin D is a secosteroid which functions through vitamin D receptor (VDR), a transcription factor, and directly or indirectly controls more than 200 heterogeneous genes including the genes for the regulation of cellular differentiation, proliferation, and angiogenesis [7]. Vitamin D is not only a pivotal mediator in various physiological processes but also plays a key role in many chronic diseases [8]. Recent studies suggest that vitamin D deficiency could adversely affect cardiovascular function, but evidences from longitudinal studies are lacking [9]. Vitamin D receptors are distributed in a variety of tissues including cardiomyocytes, vascular smooth muscle cells (VSMCs), endothelium, and cells of immune system [9]. Indeed, calcitriol, the active form of vitamin D, as well as other VDR ligands inhibit proliferation of VSMCs [10]. Vitamin D is a potent micronutrient that can immunomodulate many human diseases mediated by local autocrine or paracrine synthesis of calcitriol [11]. A number of extrarenal tissues, including VSMCs, express CYP27B1 (1-α hydroxylase) enzyme [12], which converts primary circulating form of vitamin D, 25(OH) D, to active form calcitriol. The growth suppressant and immunomodulatory effects of calcitriol are of great interest because of their potential use in the management of disorders, including post-interventional restenosis, atherosclerosis and post-transplant vasculopathy in which the underlying pathological mechanisms are uncontrolled cell growth and remodeling in the vascular wall.

In this study, we investigated the role of VDR in neointimal hyperplasia by examining the expression of TNF-α and VDR in the intimal thickening of post-interventional swine coronary arteries. The effect of vitamin D deficiency on the development of coronary artery restenosis was examined. We also investigated the effect of calcitriol on cell proliferation in porcine coronary artery smooth muscle cells (PCASMCs). Further, we assessed the effect of TNF-α on the activity and expression of VDR in PCASMCs.

## Materials and Methods

### Porcine Model of Neointimal Hyperplasia

Institutional Animal Care and Use Committee of Creighton University approved the research protocol (IACUC protocol # 0831). Animals were housed in Animal Resource Facility of Creighton University, Omaha, NE and cared according to NIH standards and USDA guidelines. Swine model of coronary artery intimal hyperplasia was developed as established [13]. Briefly, Yucatan miniswine, weighing 30–40 lbs, were purchased from Lonestar Laboratories (Sioux City, IA). Animals were fed special high cholesterol diet (Harlan Laboratories). After 6 months, animals were subjected to percutaneous transluminal balloon angioplasty (PTCA) in left anterior descending artery (LAD) or left circumflex artery (LCX). After 4-months of interventional procedures, coronary angiograms were performed followed by the sacrifice of the animal.

### Vitamin D- Deficient hypercholesterolemic Swine Model

Yucatan miniswine were divided into 2 groups of 4 animals in each group. Group 1 swine were fed with 1–1.5 lb/swine/day of a vitamin D-sufficient high cholesterol diet (Harlan, USA) with the following major ingredients: 37.2.% corn (8.5% protein), 23.5% soybean meal (44% protein), 20% chocolate mix, 5% alfalfa, 4% cholesterol, 4% peanut oil, 1.5% sodium cholate, and 1% lard. Animals in group 2 received vitamin D-deficient high cholesterol swine diet (Harlan, USA) with the following major ingredients: 19% casein “vitamin free”, 23.5% sucrose, 23.9% corn starch, 13% maltodextrin, 4% soybean oil, 4% cholesterol, and 10% cellulose. Venous blood (10 ml) from the ear vein was drawn every 8 weeks to examine complete blood count (CBC), complete metabolic profile, complete lipid profile, and serum 25(OH)D levels. Animals were housed in the Creighton Animal Facility under controlled conditions, 12∶12-h light-dark cycle at 20–24°C, without exposure to sunlight and fed a controlled diet to avoid any variation in the 25(OH) D levels due to season or diet. At 24 weeks, PTCA was performed in LAD or LCX. After 48 weeks, animals were euthanized.

### Histology and Immunohistochemistry

The heart was surgically removed and coronary arteries were carefully dissected, embedded in paraffin, and sectioned (5 µm). Histological and immunohistochemical studies were performed on paraffin sections of post-angioplasty porcine coronary arteries, as recently described [13]. The following antibodies were used: anti-smooth muscle α-actin (α-SMA) (SantaCruz biotech; sc-58669), anti- TNF-α (Abcam; Ab6671), anti-VDR (SantaCruz biotech; sc-13133), anti-proliferative cell nuclear antigen (PCNA) (Santa Cruz biotech; sc-25280). Sections incubated without the primary antibody served as negative controls. The stained sections were observed under a light microscope and pictures were taken using Olympus DP71 camera. To perform morphometric analysis, area within the lumen (LA) and within IEL was determined using NIH Image J software (http://rsb.info.nih.gov/ij/), and percent area stenosis was calculated (% area stenosis = [1-(luminal area/IEL area)]×100). Computer-assisted quantification of immunostaining was performed using NIH Image J software. Mean staining intensity from immunohistochemistry images of 3 representative sections per pig was analyzed in designated areas of selection. Five random areas were selected on the section in both media and neointima.

Proliferation of SMCs was evaluated by counting PCNA-positive cells in the neointimal regions of post-angioplasty arteries using NIH image J.

### Isolation, Culture, and Treatment of Porcine Coronary Artery Smooth Muscle Cells (PCASMCs)

PCASMCs were cultured by the established protocol in our laboratory [14] and used in passages 3–5 for *in-vitro* experiments. Prior to stimulation experiments, PCASMCs were brought to quiescent state by culturing in serum-free Dulbecco's modified eagle's medium (DMEM) (Sigma) for 24 hr. PCASMCs were treated with different concentrations (0.1–100 nM) of calcitriol (Sigma) unless mentioned otherwise.

### RNA Extraction, Reverse Transcription, and Real-Time Quantitative PCR

Total RNA was isolated using the Trizol reagent (Sigma) method. The yield of RNA was quantified by Nanodrop (GE Healthcare) and subjected to reverse transcription using the Improm-II reverse transcription kit (Promega). Then, c DNA was subjected to qPCR for VDR gene using SYBR Green PCR kit (Promega). Quantification was done by normalization against GAPDH. Following primer sequences were used: VDR, forward, 5′- AGGCTTCTTCAGACGGAGCATGAA; VDR, reverse, 5′- ACTCCTTCATGCCGATGTCCA; GAPDH, forward, 5′-GCAAAGTGGACATTGTCGCCATCA; GAPDH, reverse, 5′- TCCTGGAAGATGGTGATGGCCTTT.

### Western Blotting

Western blot was performed as described previously [13]. Briefly, each sample containing 30 µg of protein was loaded on 10–20% polyacrylamide gels and transferred to a nitrocellulose membrane (BioRad). Membranes were incubated overnight at 4°C in 5% non-fat dry milk containing VDR antibody. Bound primary antibody was detected by secondary horseradish peroxidase- conjugated antibody (Novus Biologicals). Density of the protein bands was quantified by densitometric analysis and results were normalized against GAPDH.

### Thymidine Incorporation Assay

[^3^H]-Thymidine incorporation assay for cell proliferation was done as per following protocol. PCASMCs were plated at 5×10^4^ cells/well in a 24-well plate, quiesced and stimulated with calcitriol (0.1 nM–100 nM) in SMCM with 10% FBS. After 24 hr, [^3^H]Thymidine (1 µCi) (Perkin Elmer) was added to each well and cultures were incubated at 37°C for 8 hr. PCASMCs were washed with ice cold PBS followed by addition of 1 ml of ice-cold 5% trichloroacetic acid and incubated at 4°C for 30 minutes. At room temperature, 0.5 ml of 0.5N NaOH/0.5% sodium dodecyl sulphate solution was added, properly mixed and added to scintillation vials. The amount of incorporated [^3^H] thymidine was determined using a β-scintillation counter.

### In-Vitro Cell Proliferation

The BrdU incorporation assay was performed using a cell proliferation ELISA BrdU kit (Roche Applied Science) to assess PCASMCs proliferation. Quiescent PCASMCs (5×10^4^/well) in 24-well plates were treated with different concentrations of calcitriol (10^−9^M–10^−7^M)±20 ng/ml PDGF-BB (PeproTech). After 24 h, cells were labeled with 10 µM of BrdU solution and incubated for 8 hr at 37°C. The cells were dried and fixed, and the cellular DNA was denatured with FixDenat solution for 30 min at room temperature. A mouse anti-BrdU monoclonal antibody conjugated with peroxidase was added to each well and the plates were incubated again at room temperature for 2 h. Tetramethylbenzidine was added and the cells were incubated for 30 min at room temperature. Finally, the absorbance of the samples was measured by microplate reader (PerkinElmer) at 450 nm.

### Knockdown of VDR gene

For knockdown of VDR gene, PCASMCs were transfected with 10 nM specific silencing (si) RNA oligonucleotides (Ambion), or scrambled oligonucleotides (Santa Cruz Biotech) using FuGENE6 transfection reagent (Roche Applied Science) according to manufacturer's instructions. The knockdown efficiency was analyzed by Western blot analysis. The sequences of VDR-specific siRNA oligonucleotides were as follows: sense: GCUGUUUAUUUGACAGAGAtt; antisense: UCUCUGUCAAAUAAACAGCaa.

### Apoptosis Assay

Cultured PCASMCs in 25 cm^2^ flasks were treated with calcitriol (0.1–100 nM) for 24 h and apoptosis assay was performed using Annexin-V FITC Apoptosis Detection Kit (eBioscience Cat# 88-8005) as per following protocol. PCASMCs were pelleted, washed and resuspended in 1 ml diluted binding buffer. The 100 µl of the cell suspension and 5 µl of fluorochrome-conjugated Annexin-V were mixed and incubated for 12 minutes at room temp. Cells were washed and resuspended in 200 µl of diluted binding buffer followed by addition of 5 µl of propidium iodide solution. Samples were analyzed by flow cytometry (BD Biosciences) using the PE channel for propidium iodide.

### Immunocytochemistry

Quiescent PCASMCs cultured in Lab-Tek chamber slides (Thermo-Fisher Scientific) were treated with 10 ng/ml of TNF-α (PeproTech) and/or 10 nM calcitriol. PCASMCs were fixed with ice-cold 4% paraformaldehyde for 10 minutes, incubated in 0.1% Triton X-100 for 5 minutes followed by blocking with 1% bovine serum albumin for 30 min. PCASMCs were then incubated with anti-VDR antibody followed by cyanine3-conjugated secondary antibody for 1 h each. To detect filamentous actin (F-actin), PCASMCs were incubated with Alexa Fluor 488-phalloidin (Invitrogen) for 1 hr. Slides were mounted with vectashield mounting medium (Vector Laboratories) and examined under fluorescence microscope.

### Luciferase Assay

PCASMCs were seeded in 96 well plates at the density of 1.5×10^3^ cells/well and grown in SMCM supplemented with 10% FBS. After 24 h, media was replaced with opti-MEM and cells were transfected with 100 ng of reporter (VDRE-Luc plasmid), a mixture of inducible vitamin D-responsive firefly luciferase construct and constitutively expressing Renilla luciferase construct (40∶1) (SA Biosciences), and 100 ng of negative control, a mixture of non-inducible firefly luciferase reporter and constitutively expressing Renilla construct (40∶1), using FuGENE6 transfection reagent (Roche Applied Sciences) according to manufacturer's protocol. Twenty four hours after transfection cells were incubated for 24 h with 10 nM calcitriol and/or 10 ng/ml of TNF-α. Luciferase assay was performed using the Dual-Glo luciferase assay system (Promega). Firefly and Renilla luciferases were measured in the luminometer (PerkinElmer). The activity of firefly luciferase was normalized to Renilla luciferase and expressed as fold induction compared to control.

### Statistical Analysis

Data was analyzed using GraphPad Prism 5.0 biochemical statistical package (GraphPad Software, Inc.). Values of all measurements were expressed as mean ± SEM. Statistical analysis was performed using one-way ANOVA with post-hoc analysis using Tukey's test to analyze statistically significant differences between groups. Differences at p<0.05 were considered significant.

## Results

### Development of Neointimal Hyperplasia after Balloon Injury

**Figure 1:**
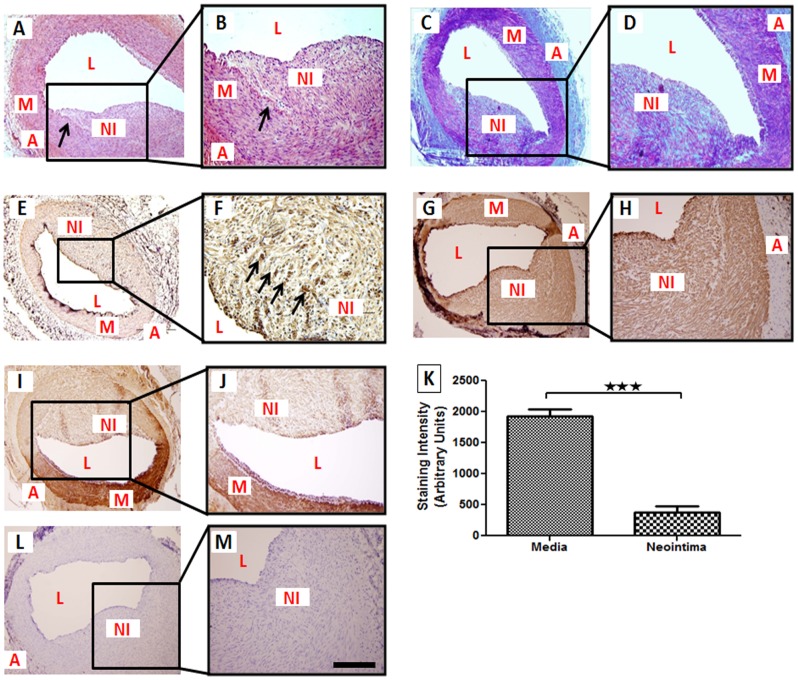
Photomicrographs of post-angioplasty swine coronary artery tissue sections. Paraffin embedded thin sections were cut, deparaffinized and histological analysis was performed using H&E, and Masson's trichrome stains and immunohistochemical analysis was done for TNF-α, VDR, and α-SMA expression. For immunohistochemistry, sections were stained with DAB as chromogen and counterstained using hematoxylin. In the H&E staining, the fracture (arrows) in the internal elastic lamina (IEL) and neointimal thickening were observed (A–B). In the Masson's trichrome staining of the tissue sections, collagen deposition in the neointimal area was prominently present(C–D). TNF-α-positive cells in post-angioplasty coronary artery are shown by arrows (E–F). The α-SMA was thoroughly expressed both in neointima and medial layer (G–H). Expression of VDR was decreased in neointima compared to media (I–J). Quantification of VDR staining in neointima and media of post-angioplasty coronary arteries was done by NIH image J (K). Two-tailed unpaired student *t* tests were performed to determine statistical relevance. Data are shown as mean ± SEM. ***P<0.001. Negative control is shown (L–M). (N = 6) scale bar 100 µm, magnification (100×–400×); A: adventitia L: lumen, M: medial layer, NI:neointima.

The histological examination by H&E staining revealed significant neointimal formation in both left anterior descending (LAD) and left circumflex arteries (LCX) 4 months following PTCA. Ball0on injury resulted in medial rupture and clear disruption of internal elastic lamina (IEL) in all balloon-injured coronary arteries (Fig. 1A–B). Masson's trichrome staining revealed abundant collagen in neointima and in adjoining adventitia (Fig. 1C–D). Percent stenosis for vessels with balloon angioplasty was 50.4±4.1%. Non-injured vessels had intact IEL without any neointimal development. Immunohistological analysis showed increased TNF-α expression in SMCs of neointimal area compared to normal SMCs in the media (Fig. 1 E–F). Immunohistochemistry was performed to examine the expression of smooth muscle α-actin (α-SMA) in neointimal area. Strong expression of α-SMA in these lesions confirmed that vascular SMCs were the main cellular component of neointimal proliferative lesions (Fig. 1 G–H). These findings were consistent across multiple samples from six different animals.

### Decreased Expression of VDR In Vivo During Intimal Thickening

Expression of VDR was examined by immunohistochemistry in balloon injured arteries. Interestingly, VDR expression was significantly decreased in the smooth muscle cells of neointimal region as compared to normal media in all tissue sections (Fig. 1I–J). VDR protein expression was quantified based on staining intensity and the data are expressed as mean ± SEM in arbitrary units (AU). Neointimal tissue showed significantly decreased (P<0.001) VDR protein expression (381.2±99.05) compared to normal media (1926±117.3) (Fig. 1K).

### Vitamin D and Lipid Profile

**Figure 2:**
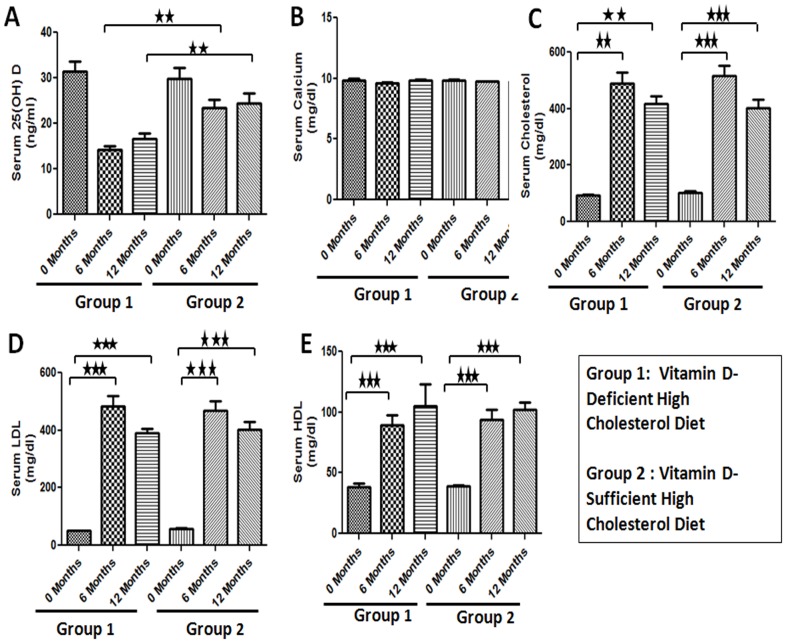
Effect of experimental diets on serum biochemical parameters in female Yucatan MiniSwine. Animals were fed vitamin D-deficient- or vitamin D-sufficient- high cholesterol diets and serum levels of 25-hydroxy vitamin D (A), calcium (B), total cholesterol (C), low density lipoprotein (LDL) (D), and high density lipoprotein (HDL) (E) were measured. Data are shown as mean ± SEM. (N = 8) **P<0.01, ***P<0.001.

To investigate the effect of vitamin D deficiency on the development of restenosis, animals were fed on vitamin D-deficient high cholesterol or vitamin D-sufficient high cholesterol experimental diets. Vitamin D-deficient high cholesterol diet produced significant vitamin D deficiency (Fig. 2A). At the time of euthanasia serum levels of 25(OH) D3, the major circulating form of vitamin D, were significantly decreased in swine on vitamin D-deficient diet (16.67±1.20 ng/ml) compared to swine on vitamin D-sufficient high cholesterol diet (24.33±2.33 ng/ml; p<.01) (Fig. 2A). Vitamin D deficiency did not have any effect on serum calcium levels (Fig. 2B). Experimental diets induced severe hypercholesterolemia in all animals. However, there was no difference in the levels of total serum cholesterol, high density lipoprotein (HDL) and low density lipoprotein (LDL) between the two groups (Fig. 2C–E)).

### Effect of Vitamin D Deficiency on the Development of Restenosis Following Balloon Angioplasty

**Figure 3:**
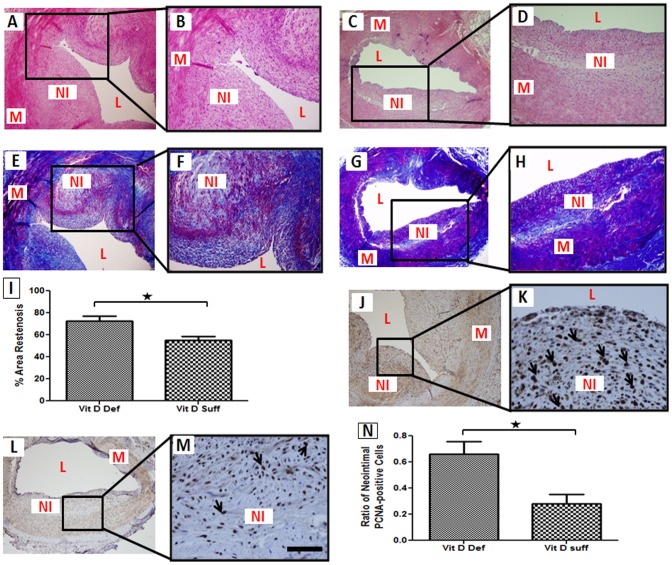
Effect of vitamin D deficiency on the development of restenosis following balloon angioplasty. Paraffin embedded thin sections were cut, deparaffinized and stained with H&E and Masson's trichrome stain and immunohistochemical examination was done for proliferating cell nuclear antigen (PCNA). For immunohistochemistry, sections were stained with DAB as chromogen and counterstained using hematoxylin. H&E staining shows the magnitude of neointimal formation in vitamin D-deficient hypercholesterolemic group (A–B) compared to vitamin D-sufficient hypercholesterolemic group (C–D). Masson's trichrome staining show increased smooth muscle cells and extracellular matrix material in neointimal tissue of vitamin D-deficient hypercholesterolemic group (E–F) compared to vitamin D-sufficient hypercholesterolemic group (G–H). Percent area restenosis was calculated by NIH image J as described under “Material and Methods” (I). Expression of PCNA in thin sections of post-balloon angioplasty coronary arteries from vitamin D-deficient hypercholesterolemic (J–K) and vitamin D-sufficient hypercholesterolemic (L–M) swine is shown. Arrows indicate cells expressing PCNA. The bar graph shows the quantitative analysis of the PCNA-positive cells counted in 5 randomly selected fields of the immunostained sections of the post-angioplasty vitamin D-deficient and vitamin D-sufficient swine coronary arteries (N). Data are shown as mean ± SEM. (N = 8) *P<0.5. Scale bar 100 µm, magnification (100×–600×); L: lumen, M: medial layer, NI: neointima.

Balloon angioplasty was performed in the coronary arteries of vitamin D-deficient or vitamin D-sufficient hypercholesterolemic pigs (4 animals per group). After a follow-up of 6-months, animals were euthanized. Development of restenosis was assessed by histomorphometric evaluation. Consistent with our pervious results from coronary injury restenosis model on histomorphometric examination, substantial neointimal formation was observed in porcine coronary arteries following balloon injury, as shown by representative H&E- and Masson's trichrome-stained sections (Fig. 3A–H). Although, the development of neointima was significant in both groups, the percent area restenosis was greater in vitamin D-deficient hypercholesterolemic group (72.48±4.48%) compared to the vitamin D-sufficient hypercholesterolemic group (54.91±3.726) suggesting that vitamin D levels affect the magnitude of restenosis development (Fig. 3I). PCNA-positive cells were increased in balloon-injured vitamin D-deficient hypercholesterolemic coronary arteries compared to vitamin D-sufficient hypercholesterolemic group (Fig. 3J–N).

### Effect of Calcitriol on the Expression of VDR

**Figure 4:**
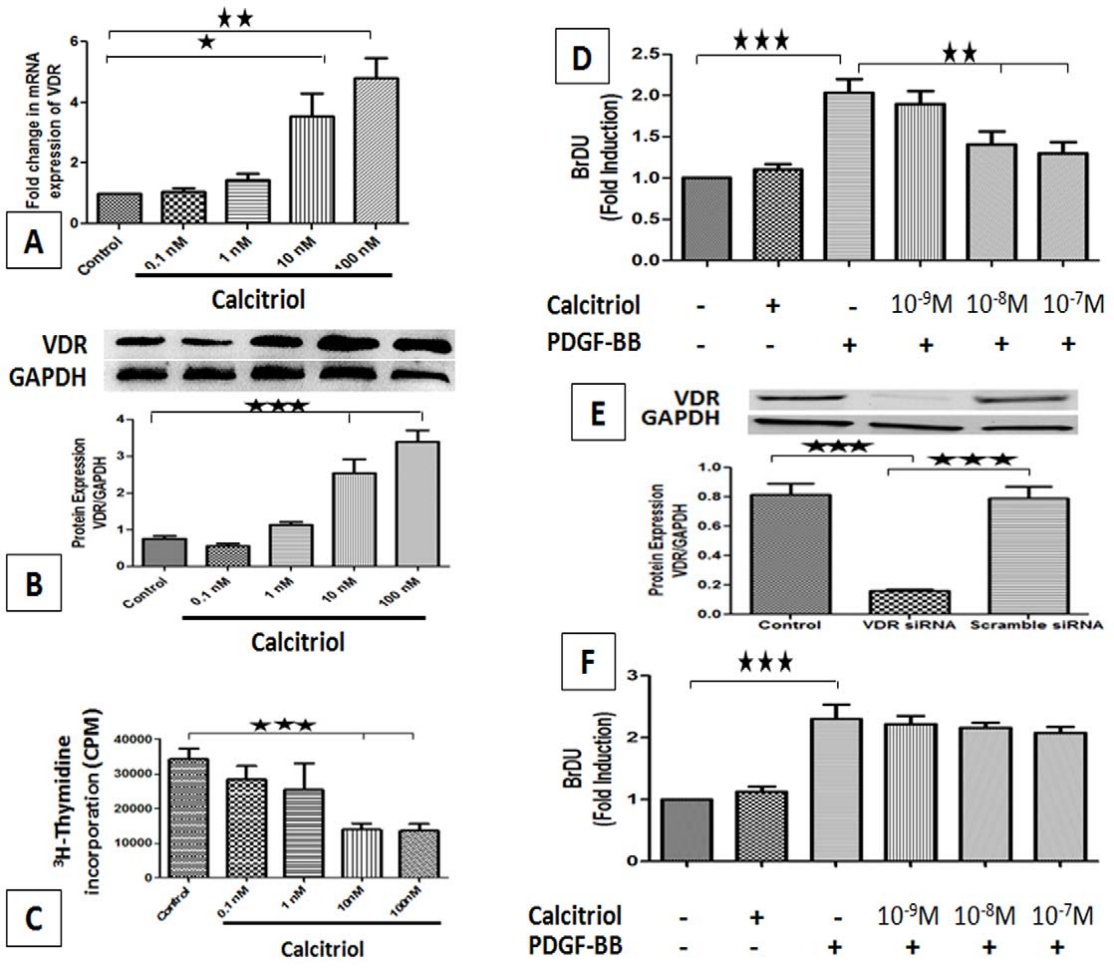
Effect of calcitriol stimulation on VDR mRNA transcript, VDR protein expression, cell proliferation in PCASMCs. Calcitriol-stimulated PCASMCs were subjected to RNA and protein isolation for qPCR (A) and Western blot (B) and data was normalized against GAPDH. Quiescent PCASMCs were incubated with calcitriol in SMCM with 10% FBS for 24 h followed by [^3^H] thymidine incorporation assay (C). Quiescent PCASMCs were stimulated with 20 ng/ml PDGF-BB with calcitriol for 24 h, and BrdU incorporation was analyzed (D). Representative Western blot and quantification of VDR protein in PCASMCs transfected with scrambled and VDR-specific siRNA (E). The relative expression of VDR protein was analyzed using GAPDH as a control. VDR knocked-down PCASMCs were stimulated with 20 ng/ml PDGF-BB with calcitriol for 24 h, and BrdU incorporation was analyzed (F). Data is mean± SEM (N = 3). *P<0.05, **P<0.01, ***P<0.001 all compared to control.

Cultured PCASMCs were incubated with various concentrations of calcitriol (0.1–100 nM). Following 24 h incubation, the mRNA transcripts and protein expression of VDR in PCASMCs was investigated by RT-PCR and western blot analysis respectively. Both RT-PCR and western blot studies showed that calcitriol stimulation significantly up-regulated the mRNA (Fig. 4A) and protein (Fig. 4B) expression of VDR gene in a dose-dependent manner.

### Effect of Calcitriol on PCASMCs Proliferation

As assessed from by [3H]-thymidine incorporation assay studies, treatment of the cells with calcitriol dose-dependently inhibited proliferation of PCASMCs following serum stimulation *in-vitro* (Fig. 4C). Proliferation of PCASMCs induced by serum was maximally inhibited (∼60%) at 10 nM and 100 nM doses of calcitriol.

### Effect of Calcitriol on PDGF-BB induced Proliferation

BrdU incorporation was measured as index of cell proliferation. Stimulation of the cells with PDGF-BB (20 ng/ml) significantly increased proliferation of PCASMCs (∼2.5 fold), but treatment with calcitriol (1–100 nM) suppressed PDGF-BB-stimulated proliferation in a dose-dependent manner (Fig. 4D).

### Effect of siRNA-induced Knockdown of VDR on PDGF-BB Induced Proliferation

To further investigate the effect of VDR on PCASMCs proliferation, we knocked down the VDR in PCASMCs by si RNA interference. As shown in Fig. 4E, transfection of the cells with VDR siRNA significantly knocked down VDR protein expression (∼80%). Interestingly, knockdown of VDR significantly abolished the effect of calcitriol on PDGF-BB-induced proliferation in PCASMCs (Fig. 4F).

### Effect of Calcitriol on PCASMCs Apoptosis

**Figure 5:**
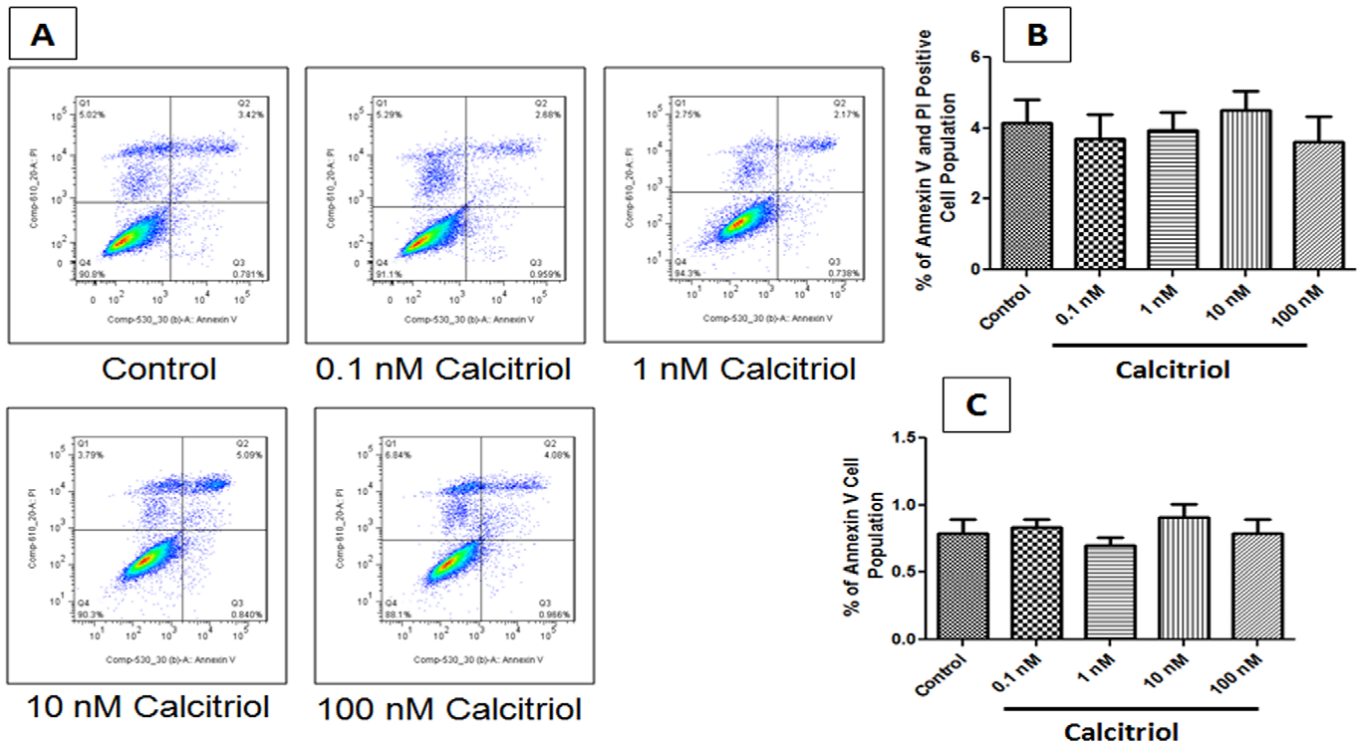
Effect of calcitriol on cell apoptosis in PCASMCs. Synchronized PCASMCs were stimulated with calcitriol stimulation (0.1–100 nM) for 24 h. (A) Dual parameter flow cytogram of FITC-labeled annexin V (Abscissa) vs PI staining (ordinate). Early apoptotic cells were labeled with annexin V only (Quadrant 3) while late apoptotic cells were positive for both annexin V and PI (Quadrant 2). Viable cells were negative both for annexin V and PI (Quadrant4); Necrotic cells were positive for PI only (quadrant 1). (B–C) Representative data of apoptosis of PCASMCs. Data shown are Mean ± SEM (N = 3).

Synchronized cultures of PCASMCs were exposed to various concentrations (0.1–100 nM) of calcitriol for 24 h. As shown in representative flow cytometric results (Fig. 5A–C), calcitriol treatment had no effect on cell apoptosis in PCASMCs with above mentioned concentrations.

### Effect of TNF-α on VDR Expression

**Figure 6:**
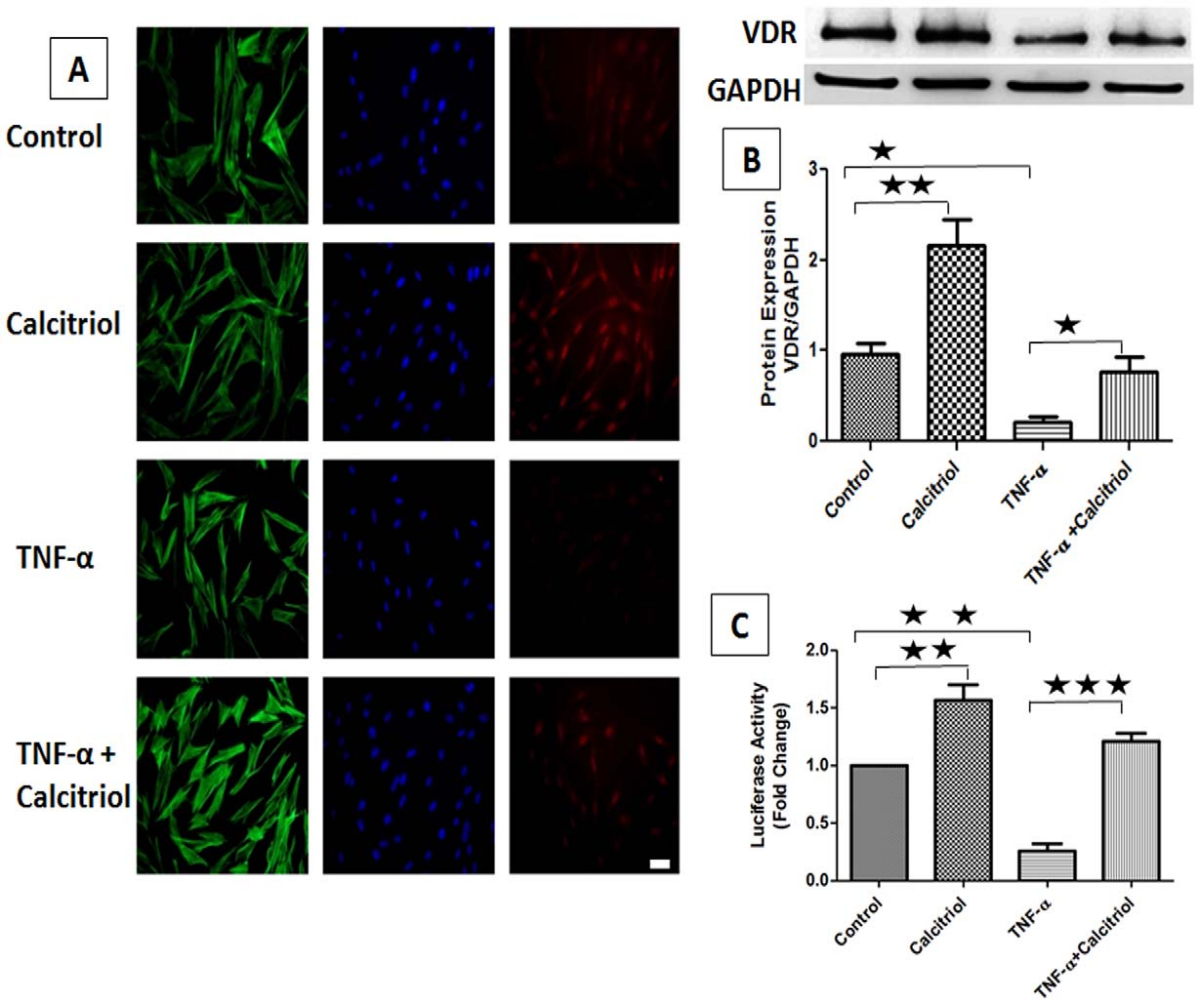
Effect of TNF-α on VDR expression in PCASMCs. (A) PCASMCs cultured in chamber slides were quiesced before addition of 10 nM calcitriol and/or 10 ng/ml TNF-α for 24 h. PCASMCs were fixed, permeabilized and stained with mouse anti-VDR and goat anti-mouse cy3 as secondary antibody. Red color indicates VDR expression and nuclei are stained blue with DAPI. PCASMCs were stained with Alexa Fluor 488 phalloidin as green for filamentous actin. Scale Bar 50 µm, Magnification 200×. (B) PCASMCs were treated with TNF-α (10 ng/ml) and/or calcitriol (10 nM) for 24 hr. Total protein was isolated followed by Western-blot analysis. Relative expression of VDR protein was analyzed against GAPDH. (C) PCASMCs transiently transfected with pVDRE-Luc were treated with 10 ng/ml TNF-α for 24 h in the absence or presence of 1onM calcitriol and subjected to luciferase assay. Fold change in relative luciferase activity was assessed relative to control. Data is shown as mean ± SEM (N = 3). *P <0.05, **P<0.01, ***P<0.001.

To ascertain whether the downregulation of VDR by TNF-α is maintained *in vitro*, PCASMCs were treated with TNF-α (10 ng/ml) and/or calcitriol (10 nM) for 24 h and protein expression of VDR was analyzed by Western blot and immunocytochemistry analysis. Calcitriol stimulation significantly upregulated VDR expression whereas TNF-α treatment significantly downregulated the protein expression of VDR. However, stimulation with calcitriol abolished the TNF-α-induced downregulation of protein expression of VDR (Fig. 6A). The findings from the immunocytochemical studies were consistent with the Western blot results (Fig. 6B).

### Effect of TNF-α on VDR Transcriptional Activity

To examine the effect of TNF-α on VDR transcription activity, VDR luciferase promoter (VDRE-Luc) was transiently expressed in PCASMCs and promoter activity was determined by luciferase assay after treatment with TNF-α for 24 h in the presence or absence of calcitriol. VDR promoter activity was significantly increased (∼60%) with calcitriol (10 nM) treatment. However, there was a significant decrease in promoter activity (∼70%) with TNF-α-stimulation, suggesting that the suppressive effect of TNF-α is due to reduced transcription of VDR gene. In addition, the TNF-α-stimulated decrease in VDR promoter activity was increased by >90% after the treatment of PCASMCs with calcitriol (Fig. 6C). These observations were consistent with the VDR protein expression levels following calcitriol and/or TNF-α stimulation (Fig. 6A–B).

## Discussion

Previous studies have demonstrated that vitamin D and its analogues are involved in the inhibition of VSMC proliferation. Most of the biological functions of vitamin D are mediated through VDR. However, to our knowledge, this is the first report on the involvement of VDR in the regulation of VSMC proliferation during intimal thickening. Here, we provide the first evidence that there is significant downregulation of VDR in proliferating SMCs of neointimal lesions *in vivo*. Additionally, we found that calcitriol inhibits proliferation in PCASMCs through VDR and TNF-α-stimulation decreases the transcriptional activity and expression of VDR in PCASMCs *in vitro*.

Angioplasty with stenting is the most common mode of treatment for CAD these days. However, restenosis is a major limiting factor following coronary intervention. Since there is no constrictive remodeling after stenting, neointimal formation plays a decisive role in restenosis after stenting [15]. Neointimal proliferation primarily consists of fibroproliferative reactions, which initially involves the proliferation and sub-intimal migration of medial SMCs [15]. With evolution of drug eluting stents (DES) and prolonged dual anti-platelet therapy, incidence of restenosis is significantly reduced with 46% reduction in the target vessel revascularization (TVR) [16], but significant concerns are still here due to rare but lethal complications like stent thrombosis [17].

Recent studies have now established that besides its classical action of maintaining calcium and phosphate homeostasis, vitamin D plays a significant role as an anti-proliferative and cell differentiating factor in different tissues [18]. VDR, a nuclear receptor, is activated upon binding of calcitriol leading to heterodimerization with retinoic acid X receptor (RXR) and this complex binds to promoter region of target genes to regulate gene transcription [19]. DNA microarray analysis of quiescent human coronary artery smooth muscle cells stimulated with VDR analogues, calcitriol and paricalcitol, has shown that both calcitriol and paricalcitol influence the VDR-mediated gene expression profile in these cells [20]. Target genes fell in the categories of cellular process, cell communication, signal transduction, development, and morphogenesis including 22 genes linked to the cardiovascular system [20].

Coronary intervention with balloon injury provokes proliferation of VSMCs resulting in neointimal hyperplasia. Swine model of coronary restenosis is widely recognized that accurately mimics the proliferative component of human restenosis [21], [22]. We observed significant neointimal development in all coronary arteries following balloon injury. TNF-α, a cytokine secreted by VSMC in the neointima, plays an important role in the pathogenesis of restenosis [23]. All post-angioplasty coronary arteries showed increased TNF-α expression in neointimal area. Interestingly, VDR expression was significantly decreased in proliferating SMCs of neointimal lesion. Both *in-vitro* and *in-vivo* studies have shown that biological response to 1, 25 (OH) D2 is directly related to the VDR content of target tissue [24], [25]. Thus, the regulation of VDR expression is vital for the hormonal actions of vitamin D. Deficiency of vitamin D has been linked with increased risk of cardiovascular disease-related mortalities including hypertension, congestive heart failure, peripheral arterial disease, and myocardial infarction [26]. However, most of these evidence come from epidemiological studies. Recent studies have also shown that vitamin D deficiency may be associated with several other indices of vascular function including development and progression of atherosclerosis [27]. However, the exact mechanism by which vitamin D might influence the development, progression and prognosis of CAD has not yet been elucidated. Additionally, it is uncertain as to what stage(s) of CAD vitamin D may have its beneficial effects. In this study, we investigated the *in-vivo* effects of vitamin D status on the development of neointimal hyperplasia following coronary intervention. Results from our study demonstrated that the development of neointimal hyperplasia after balloon injury to coronary artery negatively correlates with serum vitamin D status. The number of PCNA-positive cells in neointimal region was also significantly reduced, suggesting *in-vivo* antiproliferative effect of vitamin D.

These *in vivo* data were further confirmed in cultured PCASMCs *in vitro*. We found that VDR is present in PCASMCs at both transcriptional and translational level and stimulation of the cells with calcitriol increases VDR expression in a dose-dependent manner. These finding are in conformity with previous studies where stimulation of rat [28] or rabbit [29] VSMCs to calcitriol up-regulated VDR expression, suggesting that the effect of calcitriol on VSMCs are mediated through VDR.

The direct effect of calcitriol on the proliferation of VSMCs is not clear. In an *in-vitro* study calcitriol increased the thymidine incorporation and modulated the growth of quiescent rat VSMCs similar to α-thrombin or PDGF [30]. However, in striking contrast, this study also showed that calcitriol diminished the mitogenic response to thrombin by as much as 50% in nonquiescent rat VSMCs [30]. We observed potent anti-proliferative effects of calcitriol in PCASMCs. Interestingly calcitriol stimulation had no effect on apoptosis in PCASMCS. These findings support the study by Wu-Wong and colleagues [31] which showed that calcitriol inhibit proliferation in human coronary artery SMCs in a dose-dependent manner. PDGF-BB plays a pivotal role in VSMC proliferation and migration [32]. In this study, PDGF-BB- induced proliferation of PCASMCs was inhibited by calcitriol treatment. Such an effect of calcitriol was abolished by the specific knockdown of VDR in PCASMCs, suggesting that the growth inhibitory effect of calcitriol is mediated through VDR.

In an earlier study, TNF-α inhibited calcitriol-induced VDR activation in CV-1 cells [33]. However, it was not clear whether this effect of TNF-α was due to decreased expression of VDR. Here, for the first time, we found that the stimulation of the cells with TNF-α decreased the expression of VDR in PCASMCs. We also observed that TNF-α inhibited calcitriol-stimulated VDRE-Luc activation in PCASMCs. Additionally, TNF-α treatment also inhibits basal VDRE-Luc activation in PCASMCs. These findings suggest that TNF-α suppress VDR promoter activity in PCASMCs. Taken together, these novel findings support the results from our *in-vivo* studies.

In summary, finding from both *in-vivo* and *in-vitro* studies are suggestive of VDR downregulation by increased TNF-α concentration in neointimal VSMCs. Thus, downregulation of VDR in SMCs of post-interventional arteries due to high concentration of TNF-α could be a potentially contributing factor in the uncontrolled growth of SMCs in injured arteries leading to neointimal hyperplasia and restenosis. Anti-proliferative effect of calcitriol can prevent/decrease SMC proliferation after mechanical injury to the artery and attenuate restenosis. Consequently, we propose that vitamin D supplementation prior to coronary intervention could help in preventing the neointimal hyperplasia and restenosis, and thus, could be an inexpensive and safe therapeutic approach for reduction in cardiovascular disease burden.
